# The Oral Microbiota: Community Composition, Influencing Factors, Pathogenesis, and Interventions

**DOI:** 10.3389/fmicb.2022.895537

**Published:** 2022-04-29

**Authors:** Xinyi Li, Yanmei Liu, Xingyou Yang, Chengwen Li, Zhangyong Song

**Affiliations:** ^1^School of Stomatology, Southwest Medical University, Luzhou, China; ^2^Molecular Biotechnology Platform, Public Center of Experimental Technology, School of Basic Medical Sciences, Southwest Medical University, Luzhou, China

**Keywords:** oral microbiome, composition, biofilm, community, oral disease, microbial dysbiosis

## Abstract

The human oral cavity provides a habitat for oral microbial communities. The complexity of its anatomical structure, its connectivity to the outside, and its moist environment contribute to the complexity and ecological site specificity of the microbiome colonized therein. Complex endogenous and exogenous factors affect the occurrence and development of the oral microbiota, and maintain it in a dynamic balance. The dysbiotic state, in which the microbial composition is altered and the microecological balance between host and microorganisms is disturbed, can lead to oral and even systemic diseases. In this review, we discuss the current research on the composition of the oral microbiota, the factors influencing it, and its relationships with common oral diseases. We focus on the specificity of the microbiota at different niches in the oral cavity, the communities of the oral microbiome, the mycobiome, and the virome within oral biofilms, and interventions targeting oral pathogens associated with disease. With these data, we aim to extend our understanding of oral microorganisms and provide new ideas for the clinical management of infectious oral diseases.

## Highlights

- The communities of bacteria, fungi, and viruses in the oral cavity are discussed.
- The regulatory mechanisms linking oral microorganisms with host health and disease states are described.
- The specificity of the oral microbiota at different niches is summarized.
- Interventions targeting oral diseases caused by oral pathogens are discussed.

## Introduction

Oral microorganisms, including bacteria, archaea, fungi, viruses, and protozoa, are closely associated with oral disease processes. It has been demonstrated that the diversity of the oral microbiome in children increases after they acquire their initial colonizing microorganisms (Gomez and Nelson, 2017). A homeostatic balance is maintained between the host and the oral microbial community through a variety of bidirectional communication and regulatory mechanisms during a person’s life. However, oral infectious diseases, such as caries, periodontal disease, and oral candidiasis, can be induced by the dysbiosis of the oral microbiota (Lamont et al., 2018). The development of cancers [e.g., head and neck squamous cell cancer (Hayes et al., 2018), pancreatic cancer (Fan et al., 2018), and colorectal cancer (Flemer et al., 2018)] and various systemic diseases, including rheumatoid arthritis (Rodríguez-Lozano et al., 2019), hypertension (Czesnikiewicz-Guzik et al., 2019), Alzheimer’s disease (Liu et al., 2019), and systemic lupus erythematosus (SLE) (Pessoa et al., 2019), have been shown to be related to the oral microbiota. In the past, the limitations of traditional pure culture techniques have meant that studies of the oral microbiome focused mainly on bacteria. However, in recent years, with the development of sequencing technologies and molecular investigative methods, oral fungi, the candidate phyla radiation (CPR) group (a unique class of bacteria that co-parasitize the host), and viruses are becoming hot topics in the ecology of the human oral microbiota (Baker et al., 2017). However, until now, determining the communication and regulatory mechanisms of oral microorganisms and oral diseases has remained a challenge.

In this review, the composition of the oral microbiota and the correlations between it and oral and systemic diseases are examined. The specificity of the microbiota at different niches in the oral cavity and the interaction mechanisms between oral microorganisms and their host are also highlighted. These data should provide support for the prevention and treatment of oral diseases.

## Composition and Diversity of the Oral Microbiota

### Composition of the Oral Microbiota

There are about 1,000 species of bacteria in the oral cavity, mainly including the phyla Actinobacteria, Bacteroidetes, Chlamydia, Euryarchaeota, Fusobacteria, Firmicutes, Proteobacteria, Spirochaetes, and Tenericutes (Dewhirst et al., 2010). There are also a few lesser-known phyla and candidate divisions in the oral cavity, including Chloroflexi, Chlorobi, GN02, Synergistetes, SR1, TM7, and WPS-2 (Camanocha and Dewhirst, 2014). Among these, GN02, SR1, and TM7 belong to the CPR. Oral CPR members are thought to influence the oral microbial ecology by modulating the structural hierarchy and functions of the oral microbiome (Bor et al., 2019), which have been shown to correlate with oral diseases, such as periodontitis (Griffen et al., 2012) and halitosis (Takeshita et al., 2012). However, the pure culture of the CPR is difficult, and only TM7 had been cultured from the human oral cavity until November 2021 (Bor et al., 2020).

Oral archaea are far less numerous and diverse than oral bacteria. Although oral archaea were originally thought to be exclusively methanogens (Wade, 2013), many studies have detected DNA sequences of non-methanogenic bacteria in the oral cavity (Li et al., 2009; Dame-Teixeira et al., 2020). Surprisingly, archaea are currently recognized as non-pathogenic, although they have been detected in inflamed pulp tissue (Efenberger et al., 2015), in subgingival biofilms from patients with peri-implantitis and periodontitis (Aleksandrowicz et al., 2020), and in caries biofilm samples (Dame-Teixeira et al., 2020). These findings suggest that archaea are involved in the pathogenic processes of oral diseases. However, their potential pathogenicity requires further investigation.

There are approximately 100 species of fungi in the oral cavity. The common genera are *Aspergillus*, *Aureobasidium*, *Candida*, *Cladosporium*, *Cryptococcus*, *Fusarium*, *Gibberella*, *Penicillium*, *Rhodotorula*, *Saccharomycetales*, and *Schizophyllum* (Ghannoum et al., 2010; Peters et al., 2017). It has been confirmed that fungi account for 0.004% of the overall oral microorganisms and have only been detected in specimens from the hard palate, supragingival plaque, and oral rinses, possibly due to the nature of the participants and the low prevalence of fungi at other sites (Caselli et al., 2020). However, recent investigation detected a broad range of fungi in saliva samples and identified two well-demarcated genus-level community types (*Malassezia* and *Candida*) (Hong et al., 2020). It is noteworthy that oral fungal species show high interindividual variability in oral rinse samples from healthy individuals (Ghannoum et al., 2010).

The oral virome consists of eukaryotic viruses and phages (Caselli et al., 2020). These eukaryotic viruses mainly include *Anelloviridae*, *Herpesviridae*, and *Papillomaviridae* (Bottalico et al., 2011; Wylie et al., 2014), whereas the phages are more diverse and have been mainly studied because their capacity for bacterial lysis allow them to be used to treat bacterial infectious diseases (Shen et al., 2018; Chen J. et al., 2020). In one study, many oral viruses were not transient members of the oral ecosystem but persisted throughout the study period (Abeles et al., 2014). It has also been suggested that the viruses in saliva act as hosts for pathogenic genes in the oral environment (Pride et al., 2012). At present, the consensus on oral viruses is that they are personal, persistent, and gender-specific. Viral communities are also highly similar in the same environments (e.g., belong to the same family) (Robles-Sikisaka et al., 2013; Abeles et al., 2014).

### Core Oral Microbiota

The Human Microbiome Project (HMP) originally raised the question of whether a core human microbiome exists. A “core microbiome” is defined as the set of shared genes found in a given habitat in all humans (Turnbaugh et al., 2007). The term has evolved into five different types: the common core, temporal core, ecological core, functional core, and host-adapted core. These are defined according to their spatial distribution, temporal stability, ecological impact, and effects on host functions and health (Risely, 2020). Therefore, the oral core microbiome is the collection of microorganisms colonizing the oral cavity and their genomes, which are organized according to the five definitions mentioned above. Caselli et al. showed that there were no statistically significant differences in the microbiomes of the subjects enrolled in their study, supporting the idea that the oral core microbiome can be used as a reference for a definition of eubiosis (Caselli et al., 2020). A 16S rDNA analysis showed that Actinobacteria (genera *Corynebacterium*, *Rothia*, and *Actinomyces*), Bacteroidetes (genera *Prevotella*, *Capnocytophaga*, and *Porphyromonas*), Firmicutes (genera *Streptococcus* and *Granulicatella*), Fusobacteria (genus *Fusobacterium*), and Proteobacteria (genera *Neisseria* and *Haemophilus*) are predominant in the microbiomes of healthy people (Zaura et al., 2009). The oral microbiota also differs according to the oral niches and changes dynamically across life-history stages. For example, an analysis of saliva, supragingival, and mucosal plaque samples from healthy volunteers at different ages and stages of dentition revealed that the oral cavity is a highly heterogeneous ecological system containing distinct niches with significantly different microbial communities (Xu et al., 2015). Physiological hormones cause changes in *Campylobacter*, *Haemophilus*, *Oribacterium*, and *Prevotella*, during the female menstrual cycle (Bostanci et al., 2021). The oral abundances of *Neisseria*, *Porphyromonas*, and *Treponema* are also higher in pregnant women than in non-pregnant women. However, the abundances of *Streptococcus* and *Veillonella* are richer in non-pregnant women than in pregnant women (Saadaoui et al., 2021). These data suggest that the core oral microbiota should be defined based on the life-history stage of the host and its specific ecological locus in the oral cavity. The association between the core oral microbiota and host microecological homeostasis, health, and disease status can help us better understand the interactions among various oral microorganisms and between oral microorganisms and their hosts. Many studies are investigating the precise composition of the core oral microbiota to confirm the functional combinations of microorganisms involved in complex microecological and disease processes (Jiang et al., 2015; Oliveira et al., 2021). However, any individually defined core microbiota will not include all key microorganisms throughout the host’s entire life (Risely, 2020).

### Oral Microorganism Databases

In recent years, two major oral microbiome databases have been established, the Human Microbiome Project (HMP) and the Human Oral Microbiome Database (HOMD). HMP aims to characterize the ecology of human microbial communities and to analyze the roles of microorganisms in human health and diseases. It maps the metagenomics of microorganisms in five major parts of the human body: the oral cavity, nasal cavity, vagina, intestine, and skin (Integrative HMP (iHMP) Research Network Consortium, 2019). The oral microbiome is one of the research priorities of HMP. Nine specimens are collected from the oral cavity and oropharynx, including the saliva, buccal mucosa, keratinized gingiva, palate, tonsil, throat, tongue soft tissues, supragingival dental plaque, and subgingival dental plaque (The Human Microbiome Project Consortium, 2012). HOMD is a database of oral microorganisms launched in 2008 by the National Institute of Dental and Craniofacial Research (Bethesda Maryland, United States). Since then, the expanded Human Oral Microbiome Database (eHOMD) has provided comprehensive information on the bacterial species present in the human aerodigestive tract. The eHOMD already contains 775 microbial species, 687 of which are from HOMD version 14.51.

In summary, the development of DNA sequencing technologies and data analysis methods has greatly extended our understanding of the oral microbiome, mycobiome, and virome. Many studies have grossly underestimated the complexity of the human oral microbiome (Keijser et al., 2008; Caselli et al., 2020). Oral microorganisms often colonize different sites in the oral cavity and form distinct microbial communities on living or non-living surfaces. A comparative analysis of the microbiomes at different niches in the oral cavity has demonstrated the significant spatial specificity of the oral microbiota (Xu et al., 2015).

## Oral Niches Influence the Composition and Structure of Oral Microbiota

To explore the information related to the composition of oral microbiota at different oral niches and the influence of the niches on the composition and structure of the oral microbiota, the oral cavity is usually divided into several parts for sampling, mainly the saliva, hard tissue surface of teeth, soft tissue surfaces of the tongue, buccal mucosa, palate, and gingiva (Zhang et al., 2018).

### Saliva

The microbial composition of saliva includes the microorganisms shed from various oral niches. Therefore, saliva is considered to be a reservoir of the oral microbiota and also a fingerprint of the entire oral microbiota. However, recent studies have shown that the salivary microbiome does not represent the entire oral microbiome (Simón-Soro et al., 2013; Ren et al., 2017; Shi et al., 2018), but is significantly less representative than the microbiome of the supragingival plaque (Shi et al., 2018). Firmicutes are more abundant in the saliva, whereas Actinobacteria and Fusobacteria are more abundant in dental plaque (Shi et al., 2018). Further analysis has confirmed that the microbiota in oral rinse fluid samples is more representative of the entire site-specific microbiota than that in the saliva (Caselli et al., 2020). A study of the salivary microbiome in 997 members of the Qatari population found that the predominant member of the salivary microbiota was Bacteroidetes, and that *Gemella*, *Haemophilus*, *Neisseria*, *Porphyromonas*, *Prevotella*, *Streptococcus*, and *Veillonella* were the most prevalent genera (Murugesan et al., 2020). However, the microbial composition of the saliva from Japanese (Takeshita et al., 2016) and Chinese populations (Xun et al., 2018) differed from these results. These differences may be influenced by genetic, dietary, and environmental factors (Shaw et al., 2017). Nevertheless, the changes in the composition of the salivary microbiome can be used as a biomarker to monitor the prevalence and prognosis of diseases such as caries, oral cancer, and periodontal disease, and to reflect the oral and general health status (Lee et al., 2017; Yamashita and Takeshita, 2017).

Saliva is thought to greatly influence the colonization and clearance of microorganisms and to play important roles in the physical, chemical, and immunological defenses of the oral cavity (Fábián et al., 2012). It is involved in the formation of a protective liquid layer, the acquired pellicle, which participates in the microbial adhesion and colonization processes (Lynge Pedersen and Belstrøm, 2019). Saliva also contains a variety of defense proteins that are involved in the immunity of the oral mucosa, such as salivary immunoglobulins, antimicrobial peptides, lysozyme, α-amylase, mucin, peroxidases, and statherin (Fábián et al., 2012). For example, anxiety and depressive symptoms can affect the abundances of *Actinomyces*, *Fusobacterium*, *Leptotrichia*, Spirochaetaceae, and *Treponema* by regulating cortisol and C-reactive protein in the saliva (Simpson et al., 2020). Endogenous antimicrobial lipids also have antibacterial, antifungal, antiviral, and antiparasitic activities (Brasser et al., 2011; Fischer, 2020). The host-derived tRNA-derived small RNAs (tsRNAs) in saliva, which share sequence similarities with bacterial tRNAs, act as cross-domain mediators to regulate host-associated bacterial growth (He et al., 2018).

### Tooth Surfaces

The tooth surface is the only non-shedding surface in the oral cavity and therefore provides an ideal environment for bacterial growth and the formation of dental plaque. It has been shown that dental plaque has higher α-diversity, microbial richness, and evenness than samples of saliva and tongue (Ren et al., 2017). It is noteworthy that the plaque on the supragingival enamel surface is formed by the acquired pellicle (Lynge Pedersen and Belstrøm, 2019), whereas at subgingival sites, the gingival crepuscular fluid acts as a special source of nutrition, and its serum proteins combine with salivary proteins to form a unique proteinaceous film. The physical barrier effect of the gingiva also significantly reduces the oxygen tension and shear forces at subgingival sites, causing the compositions and structures of the supragingival and subgingival plaque microbiotas to differ (Costalonga and Herzberg, 2014). For example, anaerobic bacteria, including *Actinomyces*, *Fusobacterium*, and *Veillonella*, are predominantly found in the subgingival plaque (Caselli et al., 2020). The oral microbial composition on tooth surface is also influenced by the anatomy and physiology of the tooth surfaces and the perigingival area. The crown surface of the same tooth can be divided into five different areas: the occlusal or chewing surface, the proximal surface or contact point between teeth, the supragingival surface, the buccal or cheek-contacting surface, and the lingual surface proximate to the tongue (Costalonga and Herzberg, 2014). There are significant differences in the bacterial compositions at these different locations (Simón-Soro et al., 2013; Costalonga and Herzberg, 2014). For example, *Streptococcus* spp. are found on the labial surface of incisors and cuspids in 40–70% of cases, but hardly ever on the lingual surface (Fábián et al., 2012; Simón-Soro et al., 2013).

### Soft Tissue Surfaces

The structural characteristics of the oral mucosal epithelial layer mean that it is shed continuously. Although it is still continuously colonized by oral microorganisms, these microorganisms are relatively restricted (Costalonga and Herzberg, 2014). *Streptococcus* is the most abundant genus in mucosal tissue (Caselli et al., 2020). Interestingly, the tongue has a higher density and greater diversity of microorganisms than other mucosal surfaces (Zhang et al., 2018). Facultative and obligate anaerobes, including *Actinomyces*, *Porphyromonas*, *Prevotella*, *Streptococcus*, and *Veillonella*, are the main microorganisms in the tongue coating, probably because an anaerobic environment is provided by the tongue papillae (Ren et al., 2017). *Haemophilus*, *Leptotrichia*, and *Neisseria* are also abundant in tongue samples. Notably, the tongue biofilm is closely associated with halitosis (Zhang et al., 2021b).

It is worth noting that the oral mucosa, as the main anatomical barrier of the oral cavity, has both microbial immune barrier and physical barrier functions. The immunity of the oral mucosa is part of the body’s immune system, which also influences the composition of oral microorganisms (Fábián et al., 2012). Pattern recognition receptors are mainly expressed on immune cells, and their recognition of pathogen-associated molecules activates a variety of intracellular signaling pathways, which trigger proinflammatory and antimicrobial responses (Şenel, 2021). For instance, oral immune cells recognize the cell wall components of *Candida albicans*, and then exert antifungal effects (Zhou Y. et al., 2021). In a healthy host, *C. albicans* exists as a non-toxic yeast that induces little immune response (Garcia-Rubio et al., 2019). However, when it transforms into the mycelial state, it activates the interleukin 17 (IL17)/Th17 immune pathway (Gaffen and Moutsopoulos, 2020). A deficiency in the Th17 pathway is associated with fungal overgrowth, which may be one reason that immunocompromised people are more susceptible to oral candidiasis (Smeekens et al., 2014).

In response to salivary flow, mastication, chewing, and other factors, these locations in the oral cavity have different physicochemical properties, such as nutrients, pH, oxygen tension, and shear forces, which select suitable microorganisms for each oral niche. As a result, these oral niches display different compositions of oral microbiota. Furthermore, oral niches, such as the tongue and teeth, also provide physical support for the formation of oral microbial biofilms.

## Oral Microbial Biofilms

### Structure and Formation of Oral Microbial Biofilms

Oral microbial biofilms are complex ecological environments with plentiful and diverse oral microorganisms, which are associated with various oral diseases (Rath et al., 2021). Based on the acquired pellicle formed by salivary proteins, the initial colonizing bacteria, such as *Streptococcus gordonii, Streptococcus mitis, Streptococcus oralis*, and *Streptococcus sanguinis*, bind specifically to their complementary salivary receptors through their surface adhesins (Kolenbrander et al., 2010). Extracellular polysaccharides, structural proteins, cell fragments, and nucleic acids then contribute to extracellular polymeric substances (EPS) (Heller et al., 2017). Ultimately, a three-dimensional ecosystem is formed, composed of a variety of microorganisms, EPS, proteins, and lipids from food and saliva, as well as voiding and conduit systems (Bowen et al., 2018; Rath et al., 2021).

Because of its strong and extensive adhesive ability, *Fusobacterium nucleatum* establishes interdependent relationships with other members of the oral microbiota, playing an bridging role among the early and later bacterial colonizers (Kriebel et al., 2018). The adhesion ability of *F. nucleatum* mainly depends on its surface outer membrane proteins, which can be divided into the lactose inhibitory type (such as Fap2) and the amino acid inhibitory type (such as RadD and FomA) (Brennan and Garrett, 2019). RadD generally mediates colonization by Gram-positive bacteria, such as *Staphylococcus aureus*, *Streptococcus gordonii*, and *S. mutans*, and even *C. albicans* (Mutha et al., 2018; Lima et al., 2019). The coadhesion ability of a *radD*-gene-deleted *F. nucleatum* strain with *S. gordonii* was reduced. The lack of *fad-I*, a gene expressed immediately upstream from *radD*, increases the expression of *radD*, and then increases the coaggregation of *F. nucleatum* with *S. gordonii* and subsequent biofilm formation. These findings suggest that the lipoprotein Fad-I plays an important role in the regulation of RadD adhesion (Shokeen et al., 2020). Another small fusobacterial lipoprotein, Aid1, also participates in the interspecies interactions of *F. nucleatum*, especially with oral streptococci and related Gram-positive species (Kaplan et al., 2014). Moreover, the protein FomA (Puth et al., 2019), the autonomic transporter Fap2 (Silbergleit et al., 2020), and the newly discovered arginine inhibitory adhesive designated “coaggregation mediating protein A” (Lima et al., 2017) mediate the interaction between *F. nucleatum* and different oral microorganisms and also affect the formation of oral biofilms. And the biofilm thickness, stability, and architecture in five subspecies of *F. nucleatum* are varied, suggesting their different adhesion abilities and roles in biofilm formation (Muchová et al., 2022). *Corynebacterium* and *Veillonella* also have been confirmed to play a bridging role in oral biofilm development (Kriebel et al., 2018; Zhou P. et al., 2021).

During the maturation of oral microorganism biofilms, the main bacteria of the biofilms change from *Streptococcus* in the early stage to obligate anaerobes later, such as *Capnocytophaga*, *Fusobacterium*, *Porphyromonas*, and *Prevotella*, and especially *Actinomyces* (Takeshita et al., 2015). They gradually form complex multispecies structures, such as corncobs, palisades, and test tubes, which provide the best biochemical conditions for these members of the microbiota (Zijnge et al., 2010; Keijser et al., 2018). It has discovered a distinctive, multigenus consortium in the dental plaque, which shows the significance of microbial biogeography at the micron scale. The highly organized hedgehog structure mainly consist nine taxa, representing a radially organized spatial framework, in which *Corynebacterium* filaments radiate outward from near the center, with other species and their fibers as well as corncob structure around the distal tips of it (Mark Welch et al., 2016). In accordance with their biochemical characteristics, obligately anaerobic microorganisms tend to occur internally, whereas facultatively or obligately aerobic microorganisms tend to occur peripherally, and the microorganisms that consume or produce the same metabolite tend to occur in close proximity to each other (Mark Welch et al., 2016; Kriebel et al., 2018). In terms of bacterial morphologies and fluorescence intensities, subgingival plaque can be divided into four different layers: eukaryotic cells, top layer, intermediate layer, and basal layer (Zijnge et al., 2010). The micron-scale structure elaborates the roles of individual oral microorganisms and provides a comprehensive understanding of their community ecology and potential pathogenicity (Mark Welch et al., 2020). In addition, using multiscale imaging, sequencing analysis, and other techniques, recent investigation found that an alternative biofilm development process in multiple species, even containing human epithelial cells, shape polymicrobial communities at various spatial and taxonomic scales (Simon-Soro et al., 2022).

### Bacterial Interactions During Biofilm Formation

Coaggregation occurs when two paired individual bacteria are suspended in the planktonic phase. However, “coadhesion” usually refers to a situation in which one microorganism is fixed to a surface and the coadhering microorganism is suspended (Ruhl et al., 2014). Binding is extremely important in the formation of multispecies biofilms, and depends on the adhesins expressed on the surface of one bacterium and the complementary polysaccharide-containing receptors expressed by the other species (Chen et al., 2019). *S. gordonii*, which occurs widely in oral microbial biofilms, can coaggregate with *Actinomyces* (Mohammed et al., 2018), *Fusobacterium* (Mutha et al., 2018), *Streptococcus* (Choo et al., 2021), and *Veillonella* (Zhou P. et al., 2021), and plays an important role in the formation of biofilms. A molecular analysis showed that the antigen I/II (AgI/II) family proteins, with highly conserved structures and basic sequences, are detectable on the surfaces of all *Streptococcus* in the human oral cavity (Choo et al., 2021). SspA protein, a member of the AgI/II family of *S. gordonii*, recognizes a polysaccharide of *Actinomyces oris* T14V containing glucose, mannose, and galactose, which facilitates the coaggregation of the two bacteria. Significantly, this combination has a certain specificity. For instance, this polysaccharide only binds to SspB expressed by *Lactococcus lactis*, but does not bind to other *L. lactis* AgI/II family proteins (Back et al., 2015). A recent study showed that the novel intrageneric coadhesion between *S. agalactiae* and *S. mutans* is promoted by glucoside transferase B (GtfB) and GtfC (Liu et al., 2020). Another investigation showed that none of the six *Lactobacillus* strains tested, including clinical isolates from children with caries, could form biofilms *in vitro*. However, when grown with *S. mutans*, all *Lactobacillus* showed a significant increase in biofilm formation. This phenomenon was related the transfer of GtfB from *S. mutans* to the *Lactobacillu*s species (Wen et al., 2017).

Communication between the same or different species is mediated by chemical signals synthesized and secreted by bacteria. These signals can be divided into two groups. One group is related to cell density and quorum sensing (QS), and the other group involves signals produced by bacteria at various stages of growth (Jayaraman and Wood, 2008). QS is a microbial cell-to-cell communication process principally dependent upon the population density. As the population density increases, signals accumulate locally, and once a threshold concentration is reached, the autoinducer interacts with the receptor protein. When different target genes are activated, the physiological behavior of the bacteria, such as their virulence, competition, pathogenicity, and drug resistance, is altered (Abisado et al., 2018). Recent studies have mainly focused on the roles of acyl-homoserine lactones produced by Gram-negative bacteria, autoinducing peptides produced by Gram-positive bacteria, the LuxS/AI-2 QS system, and other signaling molecules (Wu et al., 2020). Autoinducer-2 (AI-2), detected in multiple oral pathogens and symbiotic bacteria, is regarded as a universal signaling molecule in interactions between bacterial species (Muras et al., 2022). A scanning electron microscopic analysis showed that the LuxS/AI-2 QS system had no effect on the morphology of *S. mutans*, but affected its growth (Yuan et al., 2021). Another investigation found that the LuxS/AI-2 QS system of *S. gordonii* influenced the structure and composition of the dual-species biofilm, formed with *S. mutans*, and its sensitivity to chlorhexidine. AI-2 synthesized *in vitro* mediated *S. mutans* biofilm formation and the expression of its virulence genes (Wang et al., 2017).

### Interactions Between Bacteria and Fungus During Biofilm Formation

*Candida albicans*, the most abundant fungus in the oral cavity, has been studied extensively (Ghannoum et al., 2010; Ellepola et al., 2019). The formation of mycelia and its transformation from yeast into mycelia are considered to be its key virulence factors (Priya and Pandian, 2020). In the oral cavity of children with severe early childhood caries, the presence of *C. albicans* significantly increases the abundance of highly acidogenic and acid-tolerant bacteria, such as *S. mutans*, *Lactobacillus*, and *Scardovia* species, and the activity of Gtfs in the dental plaque (Xiao et al., 2018a). The production of EPS is correspondingly increased, which could accommodate more caries-active *S. mutans* (Falsetta et al., 2014; Xiao et al., 2018a). An analysis of the interaction between *C. albicans* and *S. mutans* revealed that when the two strains are mixed and cultured, the carbohydrate-metabolism-related genes and proteins of *C. albicans* are significantly enhanced. The concentrations of other substances, such as mannan and glucan, also increased. These data indicate that mixed incubation can enhance fungal activity (Ellepola et al., 2019). A further investigation also showed that the GtfB secreted by *S. mutans* mainly binds to the *C. albicans* mannan layer, and that this mechanism greatly promotes the formation of the extracellular matrix and the development of mixed-species biofilms (Hwang et al., 2017).

The interactions between mixed bacteria are not unidirectional, but are interdependent and mutually beneficial. Symbiotic cooperative relationships depend to some extent on AgI/II. When *S. mutans* was cultured with *C. albicans*, the total biofilm biomass increased significantly. However, the loss of *spaP*, a gene encoding AgI/II in *S. mutans*, apparently reduced the abundance of *C. albicans* both *in vitro* and *in vivo*. Interestingly, the number of *spaP* mutant cells did not change *in vitro*, but decreased significantly *in vivo* when coinfected with *C. albicans*. These results suggest that AgI/II is required for the promotion of the two species biofilm (Yang C. et al., 2018). Another study found that the induction of SigX, an alternative sigma factor of *S. mutans* induced by QS signals, was observed in dual-species biofilms, but not in single-species biofilms (Sztajer et al., 2014). It has been demonstrated that the recognition of *C. albicans* by *S. gordonii* involves Als3 and SspB interaction, providing a new mechanism for the communication between fungi and bacteria (Silverman et al., 2010). As well as its interaction with *S. mutans*, *C. albicans* also interacts with *Actinomyces naeslundii* (Arzmi et al., 2015). Compared with single-species biofilms, the cross-kingdom dual-species biofilm formed by *A. viscosus* and *C. albicans* showed significantly enhanced cariogenic virulence (Deng et al., 2019).

In recent years, research into oral microbial biofilms has shifted from the detection and investigation of the microbes involved to the analysis of the EPS, extracellular proteins, and other matrix components. The matrix helps cells survive in their respective niches, plays a key role in meshing microbial cells, provides three-dimensional scaffolds to limit the cells’ diffusion, and ensures microenvironmental heterogeneity (Castillo Pedraza et al., 2017). In particular, the highly structured matrix in dental plaque produced by pathogenic *S. mutans* provides binding sites for other microorganisms (Bowen et al., 2018; Cugini et al., 2019). With a better understanding of the functions and effects of various matrix components, the development of matrix inhibitors has recently become a hot topic.

## Various Factors Affect the Changes in Oral Microorganisms

After the colonization of the oral cavity, oral microorganisms undergo ecological succession and gradually form stable microbial communities. However, the ecological balance is also influenced by a variety of endogenous and exogenous factors. These factors, including drug use, host lifestyle, environment, and host status, contribute to differences in disease susceptibility by influencing the composition, structure, and metabolic functions of oral microorganisms.

### Antibiotic Utilization

Antibiotics not only affect the composition and functions of the oral microbiome, but also induce specific metabolic changes during antibiotic interventions (Moraes et al., 2020). Like previous studies (Monroy-Pérez et al., 2020), a prospective cohort study found that Shannon’s diversity index was reduced by treatment with amoxicillin relative to that in the untreated group, and decreased continuously for 6 months (Menon et al., 2019). It is noteworthy that the relative abundance of Actinobacteria decreased by 60% in the antibiotic-treated group after 1 week and remained 50% lower than baseline after 6 months. The abundances of Bacteriodetes and Fusobacteria also decreased significantly. Surprisingly, the abundance of Proteobacteria increased at 1 month and *Aggregatibacter paraphrophilus* was detected at 1 month and persisted to the 3- and 6-month time points. However, no operational taxonomic units persisted up to 6 months in the control group (Menon et al., 2019). The use of amoxicillin has also been shown to reduce the abundances of *Neisseria*, *Streptococcus*, and *Veillonella* in the oral microbiota (Larsson Wexell et al., 2016; Moraes et al., 2020). In another study, a group of neonates was enriched in lipopolysaccharide biosynthesis and amino-acid-related metabolic functions after maternal antibiotic exposure, whereas the group of unexposed neonates was enriched in carbohydrate metabolic pathways (Li H. et al., 2019).

In addition to the commonly used amoxicillin, antibiotics with different pharmacological mechanisms of action also affect the oral microbiome and increase the abundance of genes associated with antibiotic resistance (Zaura et al., 2015; Moraes et al., 2020). In recent years, it has been reported that some common oral fungi are resistant to antifungal azoles (Gao et al., 2018). Another study showed that genes associated with resistance to ketoconazole and fluconazole were highly expressed in *C. albicans* isolated from patients with periodontal disease. These data suggest that these drug-resistant *Candida* strains play an important role in acute and chronic periodontal infections (Monroy-Pérez et al., 2020). However, antibiotics have a much smaller impact on the oral microbiome than on the gut microbiome (Zaura et al., 2015; Menon et al., 2019).

### Dietary Changes

To develop a diet conducive to oral health, many scholars have investigated the relationship between diet and the oral microbiota. An investigation of the oral microbiota in infants showed that breast-fed infants had a higher proportion of *Streptococcus*, whereas formula-fed infants had higher proportions of *Actinomyces* and *Prevotella*. Moreover, breast- and mixed-fed infants developed oral candidiasis at a lower frequency than infants who were fed solid food (Oba et al., 2020). A further investigation confirmed that the significant differences in the oral microorganisms of breast- and formula-fed infants were associated with the production of the antibacterial compound hydrogen peroxide (Sweeney et al., 2018). Even in adults, dietary changes can modulate the oral microbiota and prevent the development of related diseases (Anderson et al., 2020). Fiber, medium-chain fatty acids, piscine monounsaturated fatty acids, and polyunsaturated fatty acids are associated with the diversity and community structure of the oral microbiota (Hansen et al., 2018), and the intake of sugar and refined carbohydrates is associated with the abundance of oral bacteria. For instance, carbonated beverage consumption is positively related to the abundance of Bacteroidetes, Gammaproteobacteria, *Fusobacterium*, and *Veillonella* (Chumponsuk et al., 2021). It is generally accepted that the consumption of high-sugar foods provides a favorable environment for caries-associated bacteria, thereby increasing the prevalence of the disease. Sugar alcohols, such as erythritol, xylitol, and sorbitol, have been widely used as sugar substitutes for the prevention of caries. The daily consumption of these sugar alcohols was found to have a specific effect on the composition of the salivary microbiome (Štšepetova et al., 2019). Like sugar, excessive alcohol consumption can also cause an imbalance of the oral microbiome (Yussof et al., 2020). However, on the contrary, some believe that red wine and enological extracts exert some antibacterial effects (Sanchez et al., 2019), and that regular and moderate red wine consumption does not alter the overall diversity and stability of representative bacterial groups in human saliva (Barroso et al., 2015). Further research is required to confirm whether alcohol consumption is beneficial to oral health.

### Smoking

Smoking is widely believed to affect the composition of the oral microbiota. For instance, the Proteobacteria of the oral microbiome are significantly reduced, whereas Firmicutes and Actinobacteria are increased in current smokers (Wu et al., 2016). Interestingly, a study found no significant difference in terms of microbial diversity or richness between smokers and non-smokers (Al Bataineh et al., 2020), in contrast to the results of Jia et al. (2021) and Yu et al. (2017). This discrepancy may be attributable to multiple influential factors, including diet, ethnicity, and environment. However, it is clear that the effect of smoking on the oral microbiota is stable and persists for several years after quitting. Investigations have confirmed that after a certain period of smoking cessation, the overall composition of smoker’s oral microbiota is similar to that of never smokers, suggesting that quitting smoking is beneficial in restoring a healthy phenotype (Wu et al., 2016; Yang et al., 2019; Jia et al., 2021). However, the overall composition of the microbiota of former smokers who quit more than a year earlier tended to be more similar to that of current smokers than to that of never smokers (Jia et al., 2021). Recent investigations have demonstrated that the potential effects of smoking on the oral microbiome include the creation of an anaerobic environment, damage to the host’s immunity, alteration of the pH of the oral saliva and the adhesion of oral bacteria, and the antibacterial effects of the toxic substances in cigarette smoke (Wu et al., 2016; Jia et al., 2021).

### Disease States

The diseases associated with oral microorganisms involve multiple systems, including the cardiovascular system, digestive system, endocrine system, and other systems. For example, the salivary microbiome of obese patients differs from that of normal-weight hosts, and also changes during the period of weight loss (Džunková et al., 2020). However, it remains unclear whether the dietary changes or obesity itself affects the microbiome. Primary sclerosing cholangitis is a chronic, progressive cholestatic liver disease, characterized by progressive inflammation and fibrosis of bile ducts and multi-focal bile duct strictures, in which the salivary microbiota is significantly altered and may invade the gut (Lapidot et al., 2021). Interestingly, the relationships between systemic diseases and the oral microbiome differ. Diabetes, rheumatoid arthritis (RA), and SLE are all associated with inflammatory responses and the development of periodontal disease. One study found that the levels of *Capnocytophaga*, *Porphyromonas*, and *Pseudomonas* are elevated in diabetic patients, whereas the levels of *Prevotella* and *Selenomonas* are increased in SLE and the levels of *Leptotrichia* and *Prevotella* are increased in RA (Graves et al., 2019). It has also been reported that the development of tumors is related to changes in oral microbes (Fan et al., 2018; Flemer et al., 2018; Hayes et al., 2018). However, the mechanisms underlying the correlation between tumors and oral microorganisms are still unclear (Irfan et al., 2020). Oncological treatments can lead to changes in oral microorganisms. For instance, *Enterococcus*, *Lactobacillus*, *Staphylococcus*, *S. mutans*, and *C. albicans* increase after radiotherapy (Sami et al., 2020). Chemotherapy can also alter the composition of the oral microbiota. And the changes in bacteria are more obvious than the changes in fungi (Hong et al., 2019).

In the oral cavity, a variety of endogenous and exogenous factors can alter the abundance and composition of the oral microbial communities, leading to microbial dysbiosis, the occurrence of oral caries, periodontitis, and other diseases, and even causing systemic diseases (Sampaio-Maia et al., 2016; Valm, 2019).

## Relationships Between Oral Microorganisms and Oral Diseases

Traditionally, oral pathogens have been considered key factors in the occurrence and development of oral diseases. In recent years, based on 16S rRNA gene sequencing, it has been shown that oral diseases are not caused by a single pathogen, but by microbiotal dysbiosis (Sanz-Martin et al., 2017; Hurley et al., 2019; Qian et al., 2019). One study confirmed that the composition and structure of supragingival plaque is mainly related to caries, whereas subgingival plaque is mainly related to periodontal disease (Costalonga and Herzberg, 2014). Furthermore, when people are in a pathological state, microbial biofilms in the gingival sulcus and periodontal pocket are more likely to cause periodontal tissue inflammation (Vartoukian et al., 2009). This disordered subgingival plaque damages the immune response within the gum tissue. In turn, this compromised immune response promotes a further imbalance in host–bacterial interactions and can lead to severe microbiotal dysbiosis (Johnston et al., 2021).

*Streptococcus mutans* has long been regarded as the pathogen of caries, given its acidogenicity, acidurance, and adhesion (Baker et al., 2021). However, a study demonstrated that *S. mutans* is present in all healthy oral samples, but is not detected in all caries-affected dental samples (Bansal et al., 2020). In recent years, molecular biological detection techniques have shown that the occurrence and development of caries are also closely associated with *Actinomyces*, *Lactobacillus*, *Neisseria*, *Prevotella*, *Propionibacterium*, *Scardovia*, and even *C. albicans* and Epstein-Barr virus (EBV) (Hurley et al., 2019; Baker et al., 2021; Zheng et al., 2021). Furthermore, the dominant bacteria gradually change from high levels of *S. mutans* in the early stage to high levels of *Actinomycetes* and *Prevotella* in deep caries (Johansson et al., 2016). These data strongly support the notion that the interactions among multiple microorganisms and the dysbiosis of the entire microbial community contribute to the progression of caries. The host’s sugar consumption also determines the fate of caries by modifying the oral microbiota (Zhan, 2018). When sugar is consumed frequently, more-aciduric strains increase and the acidogenicity and acidurance of other bacteria are adaptively enhanced. The demineralization–remineralization equilibrium is disrupted by these changes (Takahashi and Nyvad, 2011). An *in vivo* investigation, based on 16S rRNA gene sequencing, also confirmed the association between sugar intake and the oral microbiotal community (Esberg et al., 2020).

Dental plaque is a key factor initiating periodontal disease (Seneviratne et al., 2011; Valm, 2019). As well as red complex bacteria (including *Porphyromonas gingivalis*, *Tannerella forsythia*, and *Treponema denticola*), *Aggregatibacter actinomycetemcomitans*, *Eubacterium nodatum*, *Filifactor alocis*, *Selenomonas sputigena*, TM7, *Treponema socranskii*, and several *Bacteroides*, *Campylobacter*, *Desulfobulbus*, and *Prevotella* species are also regarded as biomarkers of periodontitis (Abusleme et al., 2013; Galimanas et al., 2014). Recently, *Candidatus Bacteroides periocalifornicus* (Torres et al., 2019), *Catonella* (Kumar et al., 2005), TG5 (Schaumann et al., 2014), and the viral family *Redondoviridae* (Abbas et al., 2019) have also been shown to be associated with dental plaque in periodontal disease. The proportions of Bacteroidetes, Fusobacteria, Spirochaetes, and TM7 increase and those of Actinobacteria and Proteobacteria decrease as the depth of the probing pocket increases (Cai Z. et al., 2021). Interestingly, increased microbial diversity is a feature that markedly distinguishes periodontal disease from caries and other infectious diseases (Curtis et al., 2020). The hypothesis that the dysbiosis of dental plaque results from changes in the dominant species, rather than colonization by new strains, is thus further validated (Abusleme et al., 2013).

In addition to caries and periodontal diseases, other oral diseases, including oral cancer (Zhang et al., 2019), periapical periodontitis (Zargar et al., 2020), and recurrent oral ulcers (Zhu et al., 2021), are also related to changes in the oral microbiome. To identify possible representative target microorganisms, the changes in microbial diversity that occur in oral diseases are summarized in Table 1. Many investigations have confirmed that herpes simplex virus (HSV) and especially EBV play important roles in the occurrence and development of periodontitis and oral cancer (Blankson et al., 2019; Fakhry et al., 2019; Imai and Ogata, 2020). The presence of HSV can increase the virulence of *F. periodonticum*, *P. gingivalis*, *S. aureus*, and *T. forsythia*, leading to the development of generalized invasive periodontitis (Passariello et al., 2017). A recent study found that latent membrane protein 1, encoded by EBV, induces IL8 production, which promotes the progression of periodontitis in human gingival cells (Watanabe et al., 2019). However, the role of EBV in the etiology of periodontitis has not been clarified (Imai and Ogata, 2020; Tonoyan et al., 2020). In summary, the roles of viruses in oral diseases are extremely important. However, the relevant research into fungi and viruses is very limited compared with the many studies of the roles of bacteria in oral diseases.

**TABLE 1:** Oral microorganisms with varying abundances in different oral diseases.

Oral disease	Pathogens in traditional research	Oral microorganism with increased abundance from sequencing investigation	Oral microorganism with decreased abundance from sequencing investigation	References
Caries	**Genera:** *Actinomyces, Lactobacillus, Neisseria, Porphyromonas, Prevotella, Propionibacterium, Streptococcus* **Species:** *Actinomyces israelii, A. viscosus, Lactobacillus acidophilus, L. casei, L. fermentum, Streptococcus mitis, S. mutans, S. sanguinis* **Others:** *Candida albicans*	**Genera:** *Bifidobacterium, Haemophilus, Legionella, Neisseria, Prevotella, Propionibacterium, Rothia, Shuttleworthia, Veronococcus* **Species:** *Porphyromonas catoniae, Prevotella histicola, S. mutans, Veillonella dispar* **Others:** *C. albicans*, EBV	**Genera:** *Anaerosporobacter, Caldicoprobacter, Dysgonomonas, Hespellia, Proteiniphilum*, **Species:** *Capnocytophaga granulosa, Leptotrichia buccalis*,	Xiao et al., 2018b; Hurley et al., 2019; Baker et al., 2021; Zheng et al., 2021
Periodontal disease	**Genera:** *Fusobacterium, Parvimonas, Prevotella* **Species:** *Actinobacillus actinomycetemcomitans, A. viscosus, P. gingivalis, Tannerella forsythia, Treponema denticola*, **Others:** Cytomegalovirus, EBV, HSV-1	**Genera:** *Desulfobulbus, Eubacterium, Filifactor, Fretibacterium, Parvimonas, Porphyromonas, Prevotella, Tannerella, Treponema*, **Species:** *Filifactor alocis, P. denticola, P. endodontalis, P. gingivalis, T. denticola, T. forsythia* **Others:** Redondoviridae	**Genera:** *Actinomyces, Capnocytophaga, Corynebacterium, Neisseria, Rothia, Streptococcus* **Species:** *C. gingivalis, C. ochracea, Neisseria subflava, P. catoniae*, *Rothia aeria, S. infantis, S. mitis, S. oralis, S. sanguinis*	Abusleme et al., 2013; Galimanas et al., 2014; Chen et al., 2018; Pérez-Chaparro et al., 2018; Ikeda et al., 2020
Pulp periapical disease	**Genera:** *Actinomyces, Bacteroides, Enterococcus, Fusobacterium, Peptostreptococcus, Porphyromonas, Prevotella, Saccharomycodes, Streptococcus* **Species:** *Enterococcus faecalis, Peptostreptococcus micros*, *P. endodontalis, P. gingivalis*, *P. melaninogenicus*	**Genera:** *Aggregatibacter, Fusobacterium, Lactobacillus, Peptostreptococcus, Porphyromonas, Prevotella, Schwartzia, Slackia, Treponema* **Species:** *Dialister invisus*, *E. faecalis, Fusobacterium nucleatum, P. gingivalis, P. micros*, *T. denticule* **Others:** *C. albicans*, HSV	**Genera:** *Acinetobacter, Actinomyces, Corynebacterium, Granulicatella, Haemophilus, Leptotrichia, Staphylococcus, Streptococcus* **Species:** *N. subflava, P. melaninogenica, P. nanceiensis, R. mucilaginosa*	Dingsdag et al., 2016; Qian et al., 2019; Zheng et al., 2019; Zargar et al., 2020; Cai W. Y. et al., 2021
Oral cancer	**Species:** *F. nucleatum, P. gingivalis* **Others:** HPV	**Genera:** *Aggregatibacter, Alloprevotella, Capnocytophaga, Fusobacterium, Parvimonas, Peptostreptococcus, Porphyromona, Prevotella, Treponema* **Species:** *Catonella morbi, F. nucleatum, F. periodonticum, Haemophilus influenza, P. intermedia, Parvimonas micra, S. constellatus, T. alocis, T. denticola* **Others:** *Candida, Gibberella*	**Genera:** *Acitinomyces, Haemophilus, Lautropia, Porphyromonas, Rothia, Streptococcus, Veillonella* **Species:** *A. odontolyticus, H. parainfluenzae, P. pasteri, S. mitis, S. oralis, V. parvula*	Zhao et al., 2017; Yang C. Y. et al., 2018; Zhang et al., 2019
Recurrent oral ulcer	**Genera:** *Streptococcus* **Species:** *Helicobacter pylori, S. sanguinis*	**Genera:** *Actinobacillus, Alloprevotella, Fusobacterium, Haemophilus, Porphyromonas, Prevotella, Vibrio* **Species:** *C. gingivalis, C. sputigena, Escherichia coli, F. nucleatum, H. parahaemoliticus, H. parainfluenzae, N. flavescens, N. sicca* **Others:** *C. albicans, Malassezia*	**Genera:** *Streptococcus, Veillonella* **Species:** *Gemella haemolysans, S. oralis, S. salivarius, V. dispar* **Others:** *Cladosporium sp.*	Kim et al., 2016; Stehlikova et al., 2019; Yang et al., 2020; Zhu et al., 2021
Peri-implantitis	**Genera:** *Fusobacterium, Parvimonas, Staphylococcus* **Species:** *P. gingivalis, T. denticola*,	**Genera:** *Eubacterium, Filifactor, Fretibacterium, Porphyromonas, Tannerella, Treponema* **Species:** *A. cardiffensis, E. minutum, Eubacterium infirmum, Fretibacterium fastidiosum, G. sanguinis, Kingella denitrificans*, *L. hofstadii, P. gingivalis, P. intermedia, T. alocis, T. denticola, T. forsythia, T. maltophilum*,	**Genera:** *Actinomyces, Haemophilus, Rothia, Streptococcus, Veillonella* **Species:** *A. cardiffensis, E. infirmum, R. dentocariosa, S. sanguinis, V. dispar*	Zheng et al., 2015; Sanz-Martin et al., 2017; Ghensi et al., 2020

## Interventions Targeting Oral Diseases Caused by Oral Pathogens

### Antibiotic Treatments

Oral antibiotics specifically directed against oral caries can disrupt and destroy the signaling systems of pathogenic bacteria (Cui et al., 2019). For example, sulfated vizantin inhibits the extracellular release of cell-free glucosyltransferase, enhances the accumulation of cell-associated glucosyltransferase, and inhibits the maturation of *S. mutans* biofilms (Oda et al., 2020). Walkmycin C inhibits the biofilm formation and acid resistance of *S. mutans* (Eguchi et al., 2011). Moreover, the combination of amoxicillin and metronidazole exerted greater antimicrobial effects on subgingival biofilms of bacterial species *in vitro* than either drug alone (Soares et al., 2015). Further clinical trials have also shown that the administration of amoxicillin and metronidazole combined with scaling and root planning (SRP) was significantly superior to SRP alone in reducing the probing depth and clinical attachment loss, as well as clinical improvement in both the subgingival region and saliva (Hagenfeld et al., 2018; Lu et al., 2021), especially in the treatment of periodontitis in type 2 diabetic patients (Cruz et al., 2021) or aggressive periodontitis (Ardila et al., 2020).

In recent years, the development of antibiotic resistance has been accelerated by the abuse of antibiotics, especially in dentistry (Loffler and Bohmer, 2017). The effectiveness of antibiotics is rapidly being eroded by drug resistance, and the discovery of other unique antibiotics that are specific to pathogens is urgently required (Singh et al., 2017).

### Periodontal Interventions

“Periodontal intervention therapy” mainly refers to supragingival and subgingival SRP in patients with periodontal disease, which remove periodontal pathogens and calculus and create a relatively healthy environment. This therapy not only affects the composition and structure of the oral microbiome, but also influences the interactions between microorganisms (Zhang et al., 2021a). Periodontal treatment with oral mechanical cleaning and adjuvant systemic antibiotics significantly reduces the abundances of *A. actinomycetemcomitans* and *F. alocis*, whereas the abundances of health-related microorganisms, such as *Streptococcus* spp., *Rothia* spp., and *Prevotella* spp. increase (Velsko et al., 2020). After a periodontal intervention, the microbial abundance and biodiversity in the dental plaque decreased, whereas those in the saliva did not change (Yamanaka et al., 2012). Although the antimicrobial effect of periodontal intervention is often non-specific, its combination with adjuvant antibiotic therapy can achieve better dental plaque control than the antibiotic alone. Therefore, periodontal intervention therapy remains an essential technique for the treatment of oral diseases.

### Photodynamic Therapy

Antimicrobial photodynamic therapy (PDT) is a technique in which the excitation of light at an appropriate wavelength is used to irradiate a photosensitizer and release energy (Lu et al., 2021). The oxygen in tissues produces unstable singlet oxygen (^1^O_2_), cytotoxic reactive oxygen, and other substances that non-specifically attack the microbiota, resulting in the rapid oxidation of bacterial lipids and ultimately bacterial death (Dobson and Wilson, 1992; Qi et al., 2019). The bacteria in dental plaque are so sensitive to PDT, making it an effective bactericidal technique. In a randomized controlled clinical trial of periodontal disease, gingival index, probing depth, clinical attachment level, and the *A. actinomycetemcomitans* and *P. gingivalis* counts were significantly lower in the test group (SRP followed by PDT) than in the control group (SRP alone), after follow-up for 1, 3, 6, and 9 months (Gandhi et al., 2019). As in that clinical trial, the combination of PDT and photobiomodulation therapy accelerated the healing process after oral mucositis, reducing the remission time from 15 to 11 days (Pires Marques et al., 2020). Clinical trials of pulpitis (da Mota et al., 2015), periapical periodontitis (Moreira et al., 2015), oral leukoplakia (Li Y. et al., 2019), peri-implantitis (Al Habashneh et al., 2015), and adverse biofilm changes caused by orthodontic brackets (Rosa et al., 2020) showed that PTD was more effective than traditional treatments. However, further clinical trials are required to establish whether PTD can be used as an alternative mechanical debridement process in the prevention of oral infectious diseases (Javali et al., 2019).

### Probiotic Therapy

Probiotics are non-pathogenic living microorganisms that have both preventive and therapeutic effects on oral infectious diseases (Zaura and Twetman, 2019). The bacteriocins released by probiotics effectively antagonize acidic dental plaque, and produce glucanase and urease after their colonization of the oral mucosa, which can counteract plaque formation and saliva acidity, respectively (Di Pierro et al., 2015). Probiotics can induce the expression of the virulence genes of *S. mutans*, including acid tolerance genes (*atpD* and *aguD*), EPS production genes (*gtfB* and *sacB*), and QS genes (*vicKR* and *comCD*), and inhibit the immunomodulatory effects of interferon γ (IFN-γ) and IL10 (Wasfi et al., 2018). Probiotics have also been shown to play an important role in the treatment of chronic periodontal disease. For example, patients with generalized chronic periodontitis treated with SRP and probiotic lozenges had significantly reduced levels of periodontal pathogenic red and orange complexes, tumor necrosis factor α (TNF-α), and IL1β (Invernici et al., 2018). Probiotics can also play a therapeutic role in patients with oral candidiasis, reducing the number of *C. albicans* (Mundula et al., 2019). Both *L. casei* and *L. rhamnosus* exert strong antifungal activity against the growth and biofilm formation of *C. albicans* but do not affect the surface roughness of denture base resin. Therefore, they are ideal probiotics for the prevention and treatment of denture-related stomatitis (Song and Lee, 2017). *Bacillus subtilis* and *S. thermophilus* also inhibit the growth and biofilm formation of *C. albicans* in a dose-dependent manner, and can be used for the treatment of oral candidiasis (Zhao et al., 2016; Azad et al., 2021). Clinical trials have shown that the oral intake of probiotics reduces the prevalence of oral candidiasis in the frail elderly (Kraft-Bodi et al., 2015). A recent study indicated that *S. parasanguinis*, a symbiotic bacterium, directly inhibits the activity of GTF, which can prevent *C. albicans* binding to glucan (Huffines and Scoffield, 2020). Although many studies have suggested that probiotics are effective in the treatment of caries, several problems must still be considered. For example, the common probiotic *Lactobacillus* also promotes the development of caries (Hurley et al., 2019; Zheng et al., 2021), and a report indicated that the oral health status was not significantly improved after the administration of probiotics (Sivamaruthi et al., 2020).

### Quorum Quenching Therapy

With the increase in antibiotic-resistant bacteria, and because the formation and development of oral biofilms are closely associated with bacterial QS, quorum quenching (QQ), which interferes with microbial communication, is another alternative treatment for oral infections (Paluch et al., 2020). Without eradicating any oral bacteria, QQ maintains the balance of the oral microflora and simply inhibits the formation of biofilms, which prevents overgrowth of *C. albicans* and *S. epidermidis* (Weiland-Brauer et al., 2019). For example, D-galactose interrupts the AI-2 QS system by interfering with the biochemical synthesis of AI-2 proteins, thereby reducing biofilm formation (Ryu et al., 2020). Furanone compounds and D-ribose are QS inhibitors that reduced bacterial infection and the destruction of periodontal tissue caused by coinfection with *F. nucleatum* and *P. gingivalis* (Ben Amara et al., 2018). The inhibition of the peptidase domain of the ATP-binding box transporter ComA effectively inhibited streptococcal QS pathway, leading to phenotypic and/or behavioral changes (Long et al., 2020). In summary, these data confirm that disrupting dental plaque formation with QS inhibitors (or QQ) is a promising method for controlling oral-biofilm-related diseases.

### Phage Therapy

Given their highly specific effects on bacterial species and their coevolution with their host bacteria, phage can kill multidrug-resistant bacteria and even effectively destroy biofilms (Baker et al., 2017; Shlezinger et al., 2017; Torres-Barcelo, 2018). Phage therapy makes use of strictly lytic phages and is regarded as a novel method for the prevention, control, and treatment of oral infectious diseases (Shlezinger et al., 2017; Harada et al., 2018). The construction of a bacteriophage T4 RNA ligase 1 (T4 Rnl1) expressing system in *S. mutans* showed that T4 Rnl1 exerts membrane-directed antimicrobial activity against *S. mutans* and affects the spatial structure of its extracellular polysaccharide (Chen J. et al., 2020; Wolfoviz-Zilberman et al., 2021). Therefore, phages can be used as a new preventive treatment for caries. In the presence of phage ϕAPCM01, the metabolic activity of the *S. mutans* biofilm was reduced after 24 h of contact and the live cells in the biofilm decreased by at least 5log cfu/ml (Dalmasso et al., 2015). Recently, novel bacteriophages in the family *Siphoviridae* were shown to cause a 70% reduction in the biomass of *F. nucleatum* biofilms and to damage their membrane structure (Kabwe et al., 2019). A selection of phages suitable for the treatment of specific pathogens can be used as an alternative therapy or in combination with broad-spectrum antibacterial agents (Shlezinger et al., 2017). At present, phages associated with *Actinobacteria* (Hatfull, 2020), *Firmicutes* (Wolfoviz-Zilberman et al., 2021), and *Fusobacteria* (Machuca et al., 2010) have been successfully used for the treatment of oral infectious diseases. Therefore, the construction of phage libraries against oral pathogens will offer excellent opportunities for more-personalized dentistry and oral microbiome engineering (Shlezinger et al., 2017). However, there have been insufficient clinical trials to confirm the feasibility of these phages.

### Other Therapies

Fluoride nanophase materials and oral hygiene products based on natural extracts have been widely used to prevent caries. Fluoride, which exerts an anti-caries effect, not only promotes the remineralization of the enamel, but also reduces carbohydrate-decomposing microorganisms and inhibits the carbohydrate fermentation pathways of the oral microbiota (Staun Larsen et al., 2018; Lopez-Lopez and Mira, 2020). It also regulates oral biofilm formation by changing the hydrophobicity and aggregation of bacteria (Lopez-Lopez and Mira, 2020). Nanoparticles (NPs) can attach to and destroy bacterial cell walls, successfully disrupt the biofilm matrix, release related ions that damage cell functions, improve the water solubility of drugs, and carry, retain, and release drugs at fixed points and scheduled times (Gupta et al., 2016; Wang et al., 2016). Moreover, binding traditional drugs to NPs protects them from pH changes and degradation by the enzymes specific to different ecological sites (Lu et al., 2016). These advantages confer potential utility on NPs in the prevention and treatment of oral diseases. To inhibit the occurrence of secondary caries, silver ions and some antibacterial agents are conventionally added to the resin and adhesive (Dias et al., 2019). In recent years, the discovery of quaternary ammonium salts has offered a new method for the prevention and treatment of secondary caries (Beyth et al., 2006). Dimethylaminododecyl methacrylate, a quaternary ammonium salt, has pH-dependent antifungal effects on *C. albicans* (Chen H. et al., 2020). As natural antibacterial agents, extracts from plants exert important effects on the oral microbiota. For instance, the essential oil of *Thymus capitatus* strongly affects the growth of *S. mutans* and *C. albicans*, and an extract of *Citrus limon* var. pompia exerts bactericidal effects on *S. mutans*, although it only weakly affects *C. albicans* (Pinna et al., 2019). Catechol inhibits the development of oral pathogens such as *A. actinomycetemcomitans*, *P. gingivalis*, and *Prevotella intermedia*, and also inhibits the growth, adhesion, and acid production of acid-producing oral *Streptococcus* (Fournier-Larente et al., 2016; Higuchi et al., 2019). These data provide promising methods for the clinical treatment of dental caries, periodontitis, pulpitis, oral mucosal diseases, and halitosis.

## Conclusion and Furture Prospects

**FIGURE 1:**
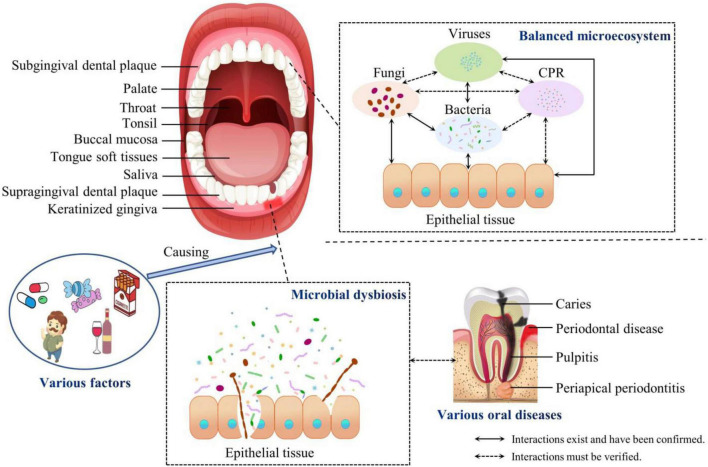
Compositions of the balanced oral microbiota and during dysbiosis. The oral cavity is divided into nine niches. The composition of the oral microbiota and the structure of the oral biofilm adapt specifically to these different microecological environments. The communities of oral microorganisms and their interactions with the host maintain the oral microecosystem in a dynamic balance. However, various factors cause the dysbiosis of the oral microbiota, which contributes to oral and even systemic diseases. CPR, candidate phyla radiation.

In this review, we have summarized recent studies of the communities of oral microbiota and the endogenous and exogenous factors that influence its composition (Figure 1). The development of metagenomics, metaproteomics, and metabonomics has provided a more comprehensive understanding of oral microbiotal communities. Increasing evidence supports the notion that various internal and external factors cause the dysbiosis of the oral microbiota, which contributes greatly to oral and systematic diseases. Therefore, more attention must be paid to the mechanisms underlying the interactions among oral microorganisms within these communities and their interactions with their host, rather than merely identifying the composition of the oral microbiome. Current studies still focus on bacteria and *C. albicans*, and identifying the communications and regulatory mechanisms within and between the mycobiome, virome, and CPR remains a challenge. We have also highlighted recent promising therapeutic strategies for oral diseases. Few investigations into the effects of these strategies on oral dysbiosis have produced consistent results, given the limitations of sampling procedures and the difficulties of enrolling subjects with heterogeneous clinical traits. To adequately mimic both the host and microbial behavior during the therapeutic process, an effective approach or model is required in which to analyze the shifts in compositions of the microbial communities.

## Author Contributions

ZS conceived and designed the manuscript. XL and YL wrote the manuscript. CL, XY, and ZS critically revised and supervised it. All authors contributed to the article and approved the submitted version.

## Conflict of Interest

The authors declare that the research was conducted in the absence of any commercial or financial relationships that could be construed as a potential conflict of interest.

## Publisher’s Note

All claims expressed in this article are solely those of the authors and do not necessarily represent those of their affiliated organizations, or those of the publisher, the editors and the reviewers. Any product that may be evaluated in this article, or claim that may be made by its manufacturer, is not guaranteed or endorsed by the publisher.
